# Treatment-Resistant Major Depression: Rationale for NMDA Receptors as Targets and Nitrous Oxide as Therapy

**DOI:** 10.3389/fpsyt.2015.00172

**Published:** 2015-12-09

**Authors:** Charles F. Zorumski, Peter Nagele, Steven Mennerick, Charles R. Conway

**Affiliations:** ^1^Department of Psychiatry, Washington University School of Medicine, St. Louis, MO, USA; ^2^Taylor Family Institute for Innovative Psychiatric Research, Washington University School of Medicine, St. Louis, MO, USA; ^3^Department of Anesthesiology, Washington University School of Medicine, St. Louis, MO, USA

**Keywords:** ketamine, suicide, hippocampus, metaplasticity, NMDA receptors, antidepressant

## Abstract

Major depressive disorder (MDD) remains a huge personal and societal encumbrance. Particularly burdensome is a virulent subtype of MDD, treatment resistant major depression (TMRD), which afflicts 15–30% of MDD patients. There has been recent interest in *N*-methyl-d-aspartate receptors (NMDARs) as targets for treatment of MDD and perhaps TMRD. To date, most pre-clinical and clinical studies have focused on ketamine, although psychotomimetic and other side effects may limit ketamine’s utility. These considerations prompted a recent promising pilot clinical trial of nitrous oxide, an NMDAR antagonist that acts through a mechanism distinct from that of ketamine, in patients with severe TRMD. In this paper, we review the clinical picture of TRMD as a subtype of MDD, the evolution of ketamine as a fast-acting antidepressant, and clinical and basic science studies supporting the possible use of nitrous oxide as a rapid antidepressant.

## Introduction

Major depressive disorder (MDD) is a common and costly psychiatric illness resulting in substantial suffering and death (1–4). While current treatments for MDD are effective, they are typically slow to work requiring weeks of administration before significant benefits are observed, and are associated with side effects that limit efficacy and patient tolerance (5). Compounding these problems, about 15–30% of patients with MDD fail to respond to available treatments (6). These latter features have prompted the need to identify faster and more efficacious antidepressant strategies.

Dating to studies initiated in the late 1990s (7), there is increasing evidence that the dissociative anesthetic, ketamine, has rapid antidepressant efficacy in subjects with MDD, including those with treatment-resistant major depression (TRMD). Ketamine differs from currently available antidepressant medications in being a non-competitive, use-dependent inhibitor of the *N*-methyl-d-aspartate class of glutamate receptors (NMDARs), and thus brings a potentially novel mechanism into play in developing new MDD treatments (8). Ketamine, however, is also a psychotomimetic, and in fact has been studied as such (9). This has prompted efforts to identify other NMDAR antagonists that have fewer side effects and more prolonged antidepressant effects compared to ketamine. Work from our labs years ago identified nitrous oxide as an NMDAR antagonist with mechanisms distinct from those of ketamine (10). Nagele et al. (11) recently conducted a pilot, proof-of-concept study using nitrous oxide (“laughing gas”) inhalation, as a potential treatment for TRMD. Although tempting to speculate that clinical benefit arises from NMDAR antagonism, nitrous oxide has other potential cellular targets that should be considered. In this paper, we will review the concepts of MDD and TRMD, the use of ketamine for TRMD, and work describing the actions and potential mechanisms of nitrous oxide as a rapidly acting antidepressant. The review is aimed at neuroscientists and those working in mental health fields.

## Major Depressive Disorder

Major depressive disorder is among the leading causes of disability, with disability defined as an inability to work productively or to live independently (3). Furthermore, MDD is a leading cause of suicide, and the presence of depression adds to the burden and adverse outcomes of primary medical illnesses, including heart disease, diabetes and cancer, among others (4). Current treatments for MDD include multiple classes of medications, a variety of neuromodulation treatments including electroconvulsive therapy (ECT), repetitive transcranial magnetic stimulation (rTMS), and vagus nerve stimulation (VNS), as well as evidence-based forms of psychotherapy such as cognitive-behavioral therapy (CBT) and interpersonal therapy (IPT).

As currently defined, MDD is a syndrome, meaning a collection of symptoms of undefined etiology that occur together over time. In the American Psychiatric Associations’ current Diagnostic and Statistical Manual of Mental Disorders-Fifth Edition (DSM-V) (12), a major depressive episode is characterized by a subset of nine clinical symptoms. These symptoms include: (1) low (sad) mood, (2) loss of interest in usual, particularly pleasurable activities, (3) change in appetite and body weight, (4) sleep disturbance, (5) dampened energy levels (fatigue), (6) increased (agitation) or decreased (retardation) motor activity, (7) feelings of excessive guilt and/or worthlessness, (8) diminished ability to focus attention and concentrate, and (9) recurrent thoughts of death and suicide.

**Figure 1:**
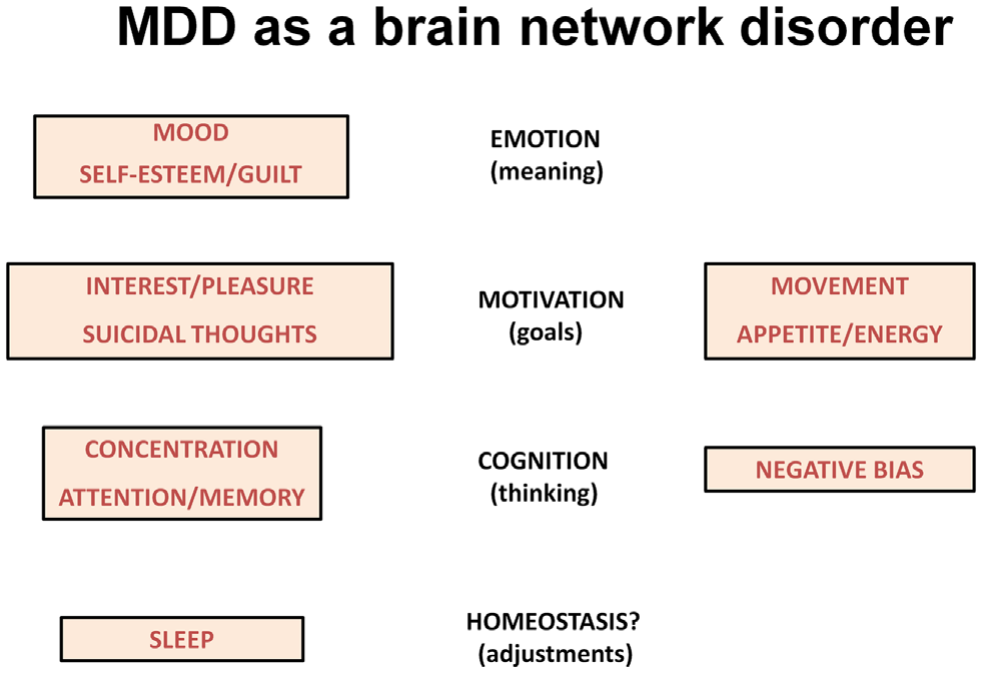
**The scheme lists symptoms of depression according to changes in brain networks underlying emotion, motivation and cognition**. Sleep disturbances may reflect homeostatic corrective efforts.

The various symptoms of MDD can be difficult to conceptualize, but include symptoms that are associated with reactions to severe and persistent stressors. An alternative way to characterize the various symptoms is to view them in terms of brain networks that are dysfunctional in major psychiatric illnesses, the networks that underlie emotional processing, motivation, and cognition (13). In this context, the low mood of depression likely reflects changes in the circuitry underlying emotional regulation (the ability to attach meaning to things and people). Certain other symptoms reflect abnormalities in cognition including the ability to focus attention and to hold items in working memory for processing (reflected in poor concentration and memory difficulties). MDD is also associated with significant changes in the brain’s motivation/reward system, the circuitry that allows humans to set goals and work toward those goals. One can interpret the problems with loss of interest in pleasurable activities, changes in appetite and energy levels, altered movements, and even suicidal ideation in this context (Figure 1).

It is important to emphasize that MDD is unlikely a single illness (13). Rather multiple illnesses of differing etiologies can produce this collection of symptoms. Furthermore, even within a given etiology, some individuals will have almost completely different symptoms, given that only five of nine symptoms are required for the MDD diagnosis. Furthermore, multiple subtypes of depression can be described depending on co-morbid conditions. Some patients with MDD have a heavy familial diathesis for MDD but do not meet diagnostic criteria for any other psychiatric disorder. In an older nomenclature, these individuals were described as having “primary” depression (sometimes called familial pure depression), meaning that MDD was the first, and in many cases, the only psychiatric disorder present (14). Similarly, individuals with bipolar disorder (BP) who experience episodes of both mania and MDD exhibit depressive symptoms that are often identical to persons who only have depression. Yet, the courses of their illness and their responses to treatments, while overlapping, have important differences. Further complicating the MDD diagnosis, individuals with psychosis (delusions and/or hallucinations) in the context of MDD require treatments that differ from MDD uncomplicated by psychotic symptoms. Similar considerations can be given for MDD arising in the context (and subsequent to the onset) of other psychiatric disorders, including substance use disorders, personality disorders, anxiety disorders, obsessive–compulsive disorder, and post-traumatic stress disorder (PTSD). Similarly, MDD occurring in the presence of major medical illnesses can have a more complicated course in which the presence of MDD and the medical illnesses bi-directionally worsens outcomes (4). The complexities noted above also raise the likelihood that there can be vastly different responses to treatment across the spectrum of MDD, and large scale clinical effectiveness trials highlight this complexity (15).

## Treatment-Resistant Major Depression

Given the heterogeneity of MDD, it is not surprising that the course of MDD can be highly variable, ranging from single episodes in some people to recurring and frequent episodes in others, and chronic unremitting symptoms in yet others. For most individuals, repeated episodes of illness are the norm with periods of illness typically ranging from several months to a year or longer. About 80% of persons with MDD have at least one recurrence during a lifetime, usually within 5 years of their initial episode (16), and individuals with an increased number of lifetime episodes require longer durations of treatment, in some cases, for life (17, 18). While current antidepressant treatments are effective, between 15 and 30% of individuals with pure MDD fail multiple treatments (1, 19–21).

Despite being relatively common in psychiatric practice, TRMD is not well defined across studies. In classifying individuals with MDD as being treatment resistant, it is important that refractoriness reflects treatment failure and not intolerance of side effects, treatment non-compliance, or inadequate dose/duration of treatment. Compounding this challenge is a problem with defining the degree of response to treatment. In clinical trials, treatment “response” is often defined as a 50% improvement in symptoms, whereas “remission” refers to having few or no residual symptoms following treatment using standardized instruments to rate symptoms including the Hamilton Depression Rating Scale (HDRS), the Montgomery–Asberg Depression Rating Scale (MADRS), and the Beck Depression Inventory (BDI), among others. Repeated studies demonstrate that remission is the goal of treatment; achieving remission gives the best chance to avoid MDD recurrence. Furthermore, “response” to treatment (50% improvement) can still reflect a state of significant dysfunction. MDD patients with multiple residual symptoms show increasing refractoriness to subsequent antidepressants trials (15).

Given the prevalence of MDD and TRMD, it is clear that TRMD is common, perhaps affecting as many as 3.5 million persons in the United States alone (1, 21). This ranks TRMD as more common than many disabling neurological conditions including epilepsy and multiple sclerosis (2). The early onset of TRMD, high medical utilization and disability also make TRMD very costly in terms of suffering, health care expenditures, and years of lost productivity.

There have been several attempts to characterize degrees of TRMD using clinical and treatment response criteria. In general, these efforts describe TRMD on an increasing scale of severity based on the number of “adequate” trials of antidepressant treatment that have been failed, with “adequate” being defined by dose and duration of the trial. In a system devised by Thase and Rush (22), TRMD is rated on a 5-point scale with the most severe degree of refractoriness involving failure of multiple medications and a course of bilateral ECT. Fava (23) described a method for staging TRMD based on rating the adequacy of dosing and duration of treatment, use of augmentation strategies (e.g., lithium, anticonvulsants, stimulants, and buspirone), and ECT. The more recent Maudsley Staging Method for TRMD provides a multidimensional system based on clinical and treatment factors (24). The latter method involves documenting the number of treatment failures including ECT and augmentation strategies while taking into account symptom severity and illness duration.

In an effort to deal with the common problem of TRMD and its complexity, Conway and colleagues (25) established the Washington University TRMD Clinic. The goal of this clinic is to provide a systematic and comprehensive evaluation and consultation program for persons with well defined TRMD, focusing primarily on patients with pure unipolar MDD. Individuals accepted into this clinic report histories of multiple, well-documented antidepressant treatment failures, but the program excludes patients with BPs, personality disorders, schizophrenia and/or schizoaffective disorder, active psychosis, or active substance use disorder (except nicotine use). Individuals in the TRMD clinic must also consent for release of all medical records so that diagnoses and treatments can be adequately characterized by chart review plus clinical diagnostic interview.

Observations on the first 79 patients evaluated in the TRMD Clinic have been reported elsewhere (25) but are instructive and relevant to the pilot clinical study of nitrous oxide described below. Patients seen in the TRMD Clinic range in age from 19 to 85 years and have an early onset of MDD, averaging 24.3 years of age at onset. This is consistent with previous studies also suggesting that TRMD has a considerably earlier mean age of onset than non-TRMD depression (17, 20, 26, 27). The TRMD clinic patients were also found to have a high rate of mood disorder in first degree relatives: (62% having a first degree relative with MDD vs. 5.1–17.5% in the general MDD population (28), and 14% having a first degree relative with BP).

**Figure 2:**
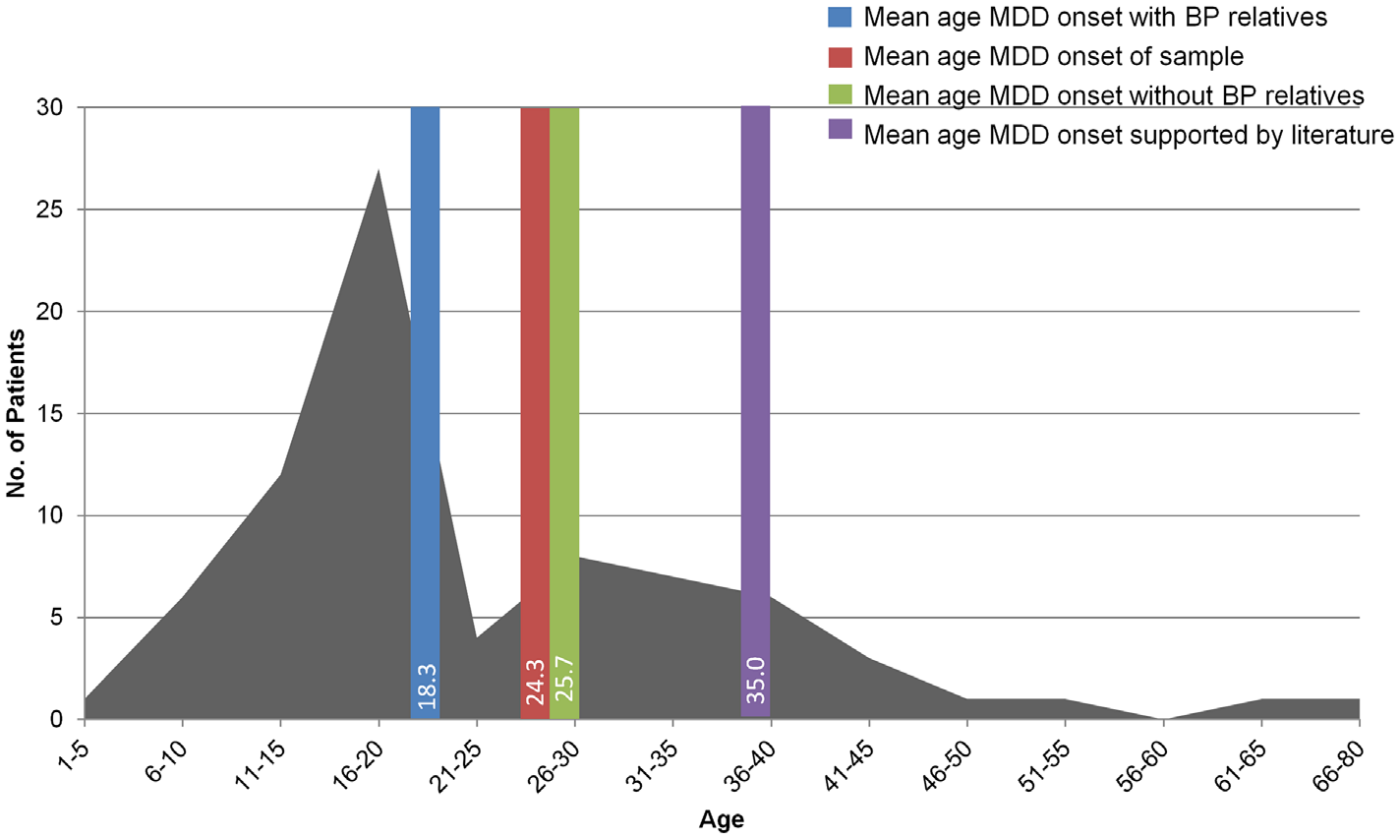
**The graph shows the age of onset of MDD in the 79 patients evaluated in the TRMD clinic**. The bars show the mean age of onset of MDD in these subjects according to family history including family members with MDD and bipolar disorder (BP). This figure has been adapted and modified from data presented in Ref. (25).

Younger age of MDD onset was associated with family history (first or second degree relative) of mood disorder (*r* = −0.26, *p* = 0.024; Figure 2) (27). Similarly, there was a statistically significant earlier mean age of MDD onset in those TRMD patients reporting a first or second degree relative with BP (mean = 18.3 years, SD = 6.7, *N* = 16) compared to those reporting no bipolar relatives (mean = 25.7 years, SD = 14.7, *N* = 62; *t* = 2.9, *p* = 0.005). This relationship supports the hypothesis that a subset of TRMD patients may represent a variant of BP. This is a potentially critical observation, as it may suggest the need for different clinical therapeutic approaches in these patients (29–33).

The TRMD clinic patients have spent almost half their lives in a depressed state: (mean of about 20 years depressed; range 5–50 years). Recurrent episodes are the norm, although 30% report one continuous episode of MDD. Based on MADRS scores, 90% of patients exhibit moderate to severe symptoms at the time of first evaluation. As supported in the literature, treatment resistance is a significant risk factor for suicide attempts: 25% have attempted suicide at some point in their life with an average of 3.4 suicide attempts per individual with an attempt (34, 35). Almost two-thirds (63%) of these patients have been hospitalized for depression and 33% are on disability. Similar to non-TRMD MDD, women outnumbered men by 2–1, consistent with other TRMD studies (16, 17, 23, 26, 27).

**Figure 3:**
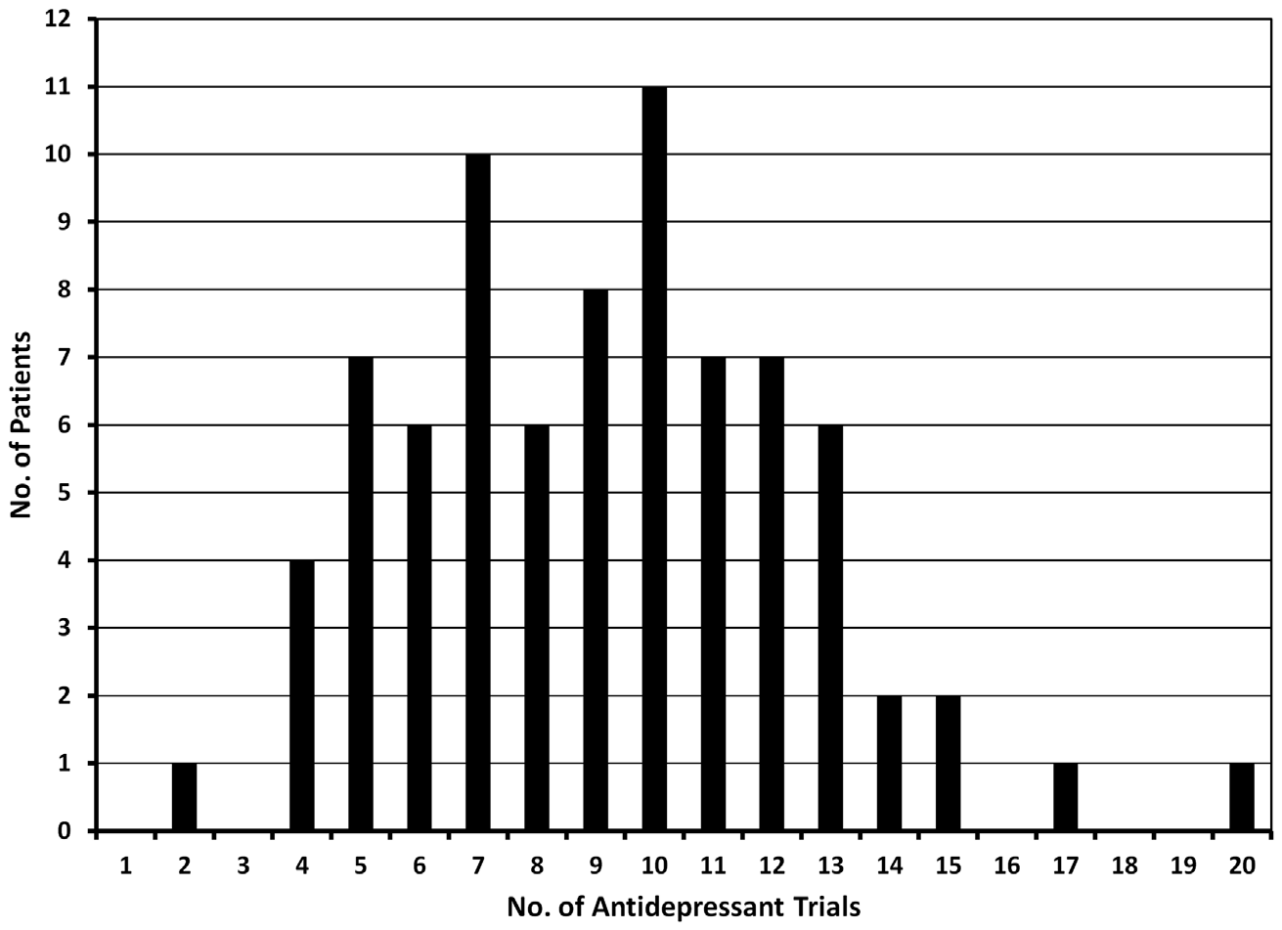
**The graph shows the number of antidepressant trials documented in 79 patients evaluated in the TRMD clinic**. Based on chart review and clinical interview, all trials listed in this graph had adequate dose and duration of antidepressant treatment.

Treatment-resistant major depression patients evaluated at the Washington University TRMD Clinic could be considered “hyper-resistant”: on average, these patients have failed 8 “adequate” treatment trials at the time of index evaluation (Figure 3). The most common treatment trials were with SSRIs (99% of patients): on average patients have received 3.6 trials of an SSRI, either alone or in combination with other treatments. SNRIs had been tried in 95% of subjects, bupropion in 89%, and evidenced-based forms of psychotherapy in 93%. Augmentation trials in which specific medications are added to ongoing treatment with a standard antidepressant were common with 86% receiving an antipsychotic (typically aripiprazole, or quetiapine), lithium (58%), stimulants (54%), thyroid supplementation (34%), and/or buspirone (23%). Interestingly, first-generation antidepressants were less commonly used with only 57% having received a TCA and 37% a MAOI. Surprisingly, the most aggressive and arguably most effective treatment for TRMD, ECT, was only attempted in 60% of these highly refractory individuals.

## Ketamine, NMDARs, and Rapidly Acting Antidepressants

The severe features of TRMD outlined above make it mandatory to find new and more effective treatments. Dating to studies initiated in the 1990s, there is increasing evidence that the dissociative anesthetic, ketamine, has rapid antidepressant effects in subjects with MDD and TRMD. Importantly, the antidepressant effects of ketamine are observed with a single intravenous administration of a subanesthetic dose, with most studies using 0.5 mg/kg infused over 40 min. In the initial pilot report, Berman et al. (7) described seven subjects with MDD who showed significant improvement in depressive symptoms within 72 h following ketamine administration. Zarate and colleagues (36) pursued this finding and reported significant benefits in 18 subjects with TRMD. These individuals showed improvement in depression ratings 2 h following ketamine infusion. By 24 h after ketamine about 70% showed a response (50% reduction in depression scores), and about 30% were in remission (having few or no symptoms). Thirty-five percent of subjects maintained the benefits 1 week after ketamine. Ketamine was fairly well tolerated although there were transient increases in psychotic symptoms during drug infusion.

The dose of ketamine used in these studies and in most studies to date is the same as originally used by Krystal and colleagues (9) in the early 1990s. In the initial studies, 19 healthy volunteers received a 40 min infusion of ketamine at a dose of 0.5 mg/kg. The purpose of these studies was to determine whether ketamine mimicked the symptoms of schizophrenia as a model to study factors involved in psychosis. The subjects experienced transient changes in perception (sensory illusions), altered thought content (persecutory ideas), amotivation (a disconnected state), and cognitive impairment (diminished attention, problems with word fluency and diminished ability to learn new information). Thus, the same dose of ketamine now being used routinely in studies of TRMD is clearly associated with psychotomimetic and cognitive effects, prompting concerns about the safety and tolerability of ketamine in individuals with psychiatric illnesses. Fortunately, in studies to date the psychotomimetic and cognitive symptoms have been transient, although ketamine also has effects on heart rate and blood pressure that may limit its use in certain patient populations. Administration of ketamine in these studies and subsequent studies was not accompanied by agents, such as GABA-enhancing agents or antimuscarinics (37), to prevent psychotomimetic side effects. It is unknown whether the use of “safening” agents would alter the antidepressant effects of ketamine.

Since the original human studies of the 1990s, work on ketamine has progressed with emphasis on defining patient groups and symptoms particularly responsive to the drug. A recent meta-analysis of seven clinical trials involving 147 patients supports the hypothesis that ketamine has rapid but transient antidepressant effects after a single administration, with odds ratios for antidepressant response and remission of 9.87 and 14.47, respectively, 24 h following treatment and effects fading over 1–2 weeks (38). A recent Cochrane review, however, was more restrained and raised cautionary notes about small sample sizes and risk of bias (39). Efforts to prolong the antidepressant effects of ketamine have included repeated infusions, following approaches used for ECT (40). For example, Murrough and colleagues (41) studied 24 subjects with TRMD treated with six intravenous ketamine infusions administered three times per week over 12 days. This group of TRMD patients showed a 71% acute response rate, with initial effects manifest within 4 h of infusion. The repeated exposures had some beneficial effect, but the median time to relapse was 18 days even with six treatments. An alternative approach to prolong the effects comes from the analgesia literature where longer ketamine infusions are used to treat chronic regional pain syndrome (CRPS). Ketamine infusions to treat CRPS have ranged from several hours to several days. For example, Dahan et al. (42) infused ketamine at a dose of 5–20 mg/h for 100 h. This prolonged treatment led to a 67% response rate (vs. 21% for placebo) and effects lasted about 50 days. Whether such an approach would be tolerated or effective in TRMD is unknown, but even longer ketamine infusions have been used elsewhere in chronic pain (43). Other efforts to prolong ketamine’s effects include intranasal administration (44).

Beyond TRMD, some evidence suggests that ketamine is effective in patients with refractory bipolar depression (45) and chronic post-traumatic stress disorder (PTSD) (46). Other studies indicate that ketamine may have beneficial effects on specific subsets of depressive symptoms including suicidal ideation (47, 48). An interesting twist is that family history may predict ketamine response. At least two studies indicate that having a positive family history of alcohol abuse/alcoholism predicts antidepressant response to ketamine (45, 49). Furthermore, prior studies indicate that recovered alcoholics and persons with high familial risk for alcoholism have altered responses to ketamine compared to naïve controls, with dampened psychotomimetic and dissociative symptoms (50–52).

## Ketamine Mechanisms

Why ketamine? The seminal studies of Krystal and colleagues (9) were designed to test the hypothesis that hypofunction of NMDARs is involved in the pathophysiology of schizophrenia and other psychotic disorders (37, 53). In parallel, Trullas and Skolnick (54) proposed that NMDAR antagonists might have antidepressant effects based on studies in animals. NMDARs are a class of glutamate-gated cationic channels in which the binding of glutamate promotes the opening of transmembrane ion channels that allow the influx and efflux of positively charged ions including Na^+^, K^+^, and Ca^2+^ (55). NMDARs are multimeric proteins with four subunits. The major NMDARs in mammalian brain express one of eight GluN1 splice variants along with one or more GluN2 subunits, or sometimes a GluN3 (GluN3A, GluN3B) subunit. GluN2 subunits (GluN2A-D) bind agonists (glutamate) in their extracellular domains, while GluN1 subunits, obligatory for a functional channel, regulate ion channel function by binding the co-agonists glycine or d-serine. Together the binding of glutamate and a co-agonist is required for opening (gating) of NMDAR ion channels (55, 56). Adding to the complexity, multiple other endogenous ions and modulators also bind NMDARs to regulate ion channel function. These include ionic modulators, Mg^2+^, Zn^2+^, and H^+^, as well as polyamines, neurosteroids, and oxysterols (55–57). Mg^2+^ plays a key role in determining the properties of NMDARs, producing a form of voltage-dependent open channel block at membrane potentials near the neuronal resting membrane potential. When the membrane depolarizes, Mg^2+^ exits the NMDAR channel and this allows other cations to flow in and out of the cell. In effect, NMDARs pass very small currents at resting membrane potential, but open effectively when neurons are excited (depolarized). This voltage-dependence contributes strongly to the proposed role of NMDARs as “coincidence detectors” because their activation requires both presynaptic (glutamate release) and postsynaptic activation (depolarization) (55, 56). When both conditions occur, NMDARs participate in excitatory synaptic function. In turn, activated NMDAR channels are highly permeable to Ca^2+^, and the influx of Ca^2+^ activates second messenger systems and promotes Hebbian synaptic plasticity, including long-term potentiation (LTP) and long-term depression (LTD), forms of lasting synaptic change thought to underlie learning and memory (58).

In the 1980s, a series of studies dating to David Lodge and colleagues (59) found that ketamine, its structural analog, phencyclidine (PCP), and the experimental agent, MK-801, are non-competitive/uncompetitive NMDAR antagonists. These agents inhibit responses mediated by NMDARs, but do not alter the binding of agonists to NMDARs unlike competitive antagonists (60). Biophysical experiments showed that ketamine and its analogs have unique mechanisms. These agents do not act on closed NMDARs. Rather, the ability of ketamine to block responses requires that NMDAR ion channels open (61, 62). When the ion channels are open, ketamine enters the channel and binds a site that is electrically deep within the channel pore to occlude the flow of ions. In contrast, memantine, another NMDAR channel blocker also binds a superficial site within the channel, perhaps accounting for differences from ketamine (63). When ketamine binds the deep site, the ion channel is able to close around the blocking molecule creating a longer-lived ion channel block. This is referred to as “trapping ion channel block” (61, 63). Relief of this block requires that glutamate (or other agonist) binds the receptor to promote channel opening. Depolarization of the neuronal membrane speeds blocker dissociation. As mentioned, ketamine shares these properties with PCP and MK-801, but is less potent than the other two agents, resulting mainly from faster dissociation from the open channel. While block of NMDARs is not the only effect of ketamine, and effects on other receptors and channels likely contribute to some pharmacological actions, the effects of ketamine on NMDARs appear to play critical roles in the ability of this agent to produce anesthetic, psychotomimetic, analgesic and possibly antidepressant effects. NMDARs are also likely to underlie the abuse potential of ketamine.

How ketamine produces its beneficial effects at cellular and molecular levels is an area of active investigation. Importantly, the effective antidepressant doses of ketamine are subanesthetic and at these concentrations, ketamine only partially inhibits NMDAR currents with responses mediated by AMPA-type glutamate receptors and unblocked NMDARs remaining intact (likely <50% NMDAR block in the presence of physiological Mg^2+^) (64–67). Studies in animals and *in vitro* indicate that ketamine has disinhibitory effects in neocortex that may result from preferential dampening of excitation of GABAergic interneurons resulting in stimulation of pyramidal (excitatory) neurons (68). Preferential inhibition of NMDARs in select cell types might be achieved through the complex dependence of ketamine block on agonist presentation, third-party endogenous modulators (e.g., positive allosteric modulators, Mg^2+^), and activity levels (64, 69, 70).

Because ketamine’s antidepressant effects greatly outlive psychotomimetic effects and systemic drug presence, it is likely that ketamine triggers persistent biochemical, synaptic and/or morphological changes. Consistent with this idea, ketamine indirectly enhances AMPAR-mediated excitatory synaptic function in frontal cortex (71) and hippocampus (72), resulting in persistent increases in synaptic efficacy and changes in neuronal structure. Other key targets for ketamine’s antidepressant effects in rodents appear to include infralimbic prefrontal cortex (73). Mechanisms contributing to synaptic enhancement may differ between regions. In frontal cortex, synaptic enhancement involves activation of the mammalian target of rapamycin (mTOR) kinase and effectors downstream of mTOR (71, 74, 75), while in hippocampus effects on eukaryotic elongation factor 2 kinase contribute (72). Other key components of ketamine-induced signaling appear to include brain-derived neurotrophic factor (BDNF) and perhaps glycogen synthase kinase 3β (72, 74). Effects of ketamine in rodent hippocampus may specifically involve effects on spontaneous synaptic transmission (72, 76).

Some evidence suggests that effects of ketamine on a specific subtype of NMDARs expressing GluN2B subunits may be particularly important in synaptic and behavioral effects in animals (77), and that activation of non-GluN2B expressing NMDARs that are not blocked by ketamine may drive some of the synaptic and metaplastic effects of ketamine on hippocampal network function (78). The latter findings are also consistent with data indicating that a selective inhibitor of GluN1/GluN2B NMDARs has antidepressant efficacy in humans (79). Other work indicates that selective blockade of NMDARs expressing either GluN2A or GluN2B has antidepressant-like effects whereas concurrent block of both subtypes results in stereotyped and possibly psychotic-like behaviors (80). It is interesting to note that these subunit selective blockers act through different pharmacological mechanisms than ketamine; they are not channel blockers. Data also indicate that activation of AMPARs, the primary mediators of fast glutamatergic transmission are important in the effects of ketamine (71, 72), but it is unclear whether this involves specific downstream effects of AMPARs or the fact that depolarization mediated by AMPARs is important for activation of unblocked NMDARs.

## Ketamine and Brain Circuits

Several studies have begun to address how ketamine affects human brain circuitry involved in cognition, motivation, and emotion. Some evidence using functional magnetic resonance imaging (fMRI) suggests that depression reflects a state of functional resting state hyperconnectivity among several brain networks including the default mode network that processes internal (self) information and networks underlying cognitive control and affective processing. Increased connectivity in these networks appears to involve increased activity in regions of dorsal medial prefrontal cortex referred to as the “dorsal nexus” and improvement in depressive symptoms is associated with dampened dorsal nexus activity (81). Scheidegger and colleagues (82) found that within 24 h after infusion of ketamine in normal subjects, there was diminished resting state connectivity of the default mode, cognitive control, and affective networks with the dorsal nexus, suggesting a plausible brain circuitry mechanism for antidepressant actions. Other work indicates that ketamine acutely dampens both the activation and deactivation of brain regions involved in a working memory task, although these latter findings may be more relevant to changes associated with schizophrenia and ketamine-induced psychotic symptoms (83, 84). Ketamine also produces acute glutamate-mediated hippocampal hypermetabolism, and this may contribute to interneuron dysfunction and acute psychotic symptoms (85).

Recent work using magnetoencephalography to map changes in regional brain interactions found that subanesthetic ketamine decreases the apparent gain of pyramidal neurons in parietal cortex, with diminished glutamate-mediated connectivity between frontal and parietal regions (86). Changes in the signal-to-noise ratio of information processing in lateral prefrontal neurons have also been observed during working memory tasks in macaques following subanesthetic ketamine (87). Taken together with studies in rodents, it appears that ketamine may enhance excitatory synaptic function in some brain regions (e.g., hippocampus and frontal cortex) while dampening excitatory connectivity in regions that are overactive in MDD (e.g., default mode, affective and cognitive control networks, and dorsal nexus).

## Other NMDAR Antagonists: Not All are Antidepressant

The clinical results with ketamine in TRMD have prompted a search for other NMDAR antagonists that share ketamine’s rapid antidepressant actions while having fewer side effects (88). These include agents with more selective actions at NMDAR subtypes, including selective GluN1/GluN2B NMDAR antagonists (79) as well as NMDAR antagonists that modulate glycine/d-serine sites on NMDARs, and others that have different effects on NMDAR channels (88–90). Although some candidate NMDAR antagonist antidepressants are not channel blockers, not all NMDAR channel blockers have significant antidepressant effects. For example, memantine, an agent used to slow progression in Alzheimer’s disease, is a non-competitive NMDAR antagonist and channel blocker with limited antidepressant efficacy (17, 91). The lack of antidepressant efficacy may result from pharmacokinetic differences and/or selective effects of memantine on NMDAR channels and subtypes (92, 93). On the other hand, differences between memantine and ketamine on NMDAR currents have been difficult to demonstrate in spontaneously active neuronal networks (70) unless third-party positive modulators of NMDARs are present (71). Thus, key pharmacological features predicting antidepressant effects are murky at the moment, and an empirical, trial-and-error approach appears warranted.

## Nitrous Oxide as a Rapidly Acting Antidepressant

Nitrous oxide is an inhalational anesthetic with analgesic and anxiolytic actions that has been in clinical use for more than 150 years (94). Acute euphorogenic effects of nitrous oxide (hence the name “laughing gas”) have also led to recreational use and abuse, and speculation that it might have antidepressant properties (95). In the late 1990s, nitrous oxide was found to be a non-competitive NMDAR inhibitor, acting by a mechanism distinct from ketamine and not involving open channel block (10, 96). Based on these early findings and evolving results from antidepressant studies of ketamine, Nagele and colleagues (11) pursued a pilot, proof of concept clinical trial using a subanesthetic dose of nitrous oxide in a group of 20 patients with TRMD. The dose used for this study was 50% nitrous oxide with 50% oxygen inhaled for 1 h in a single session. This dose was selected based on its routine use for analgesia and mild sedation in anesthesiology and dentistry. The effects of nitrous were compared to a placebo gas mixture (50% nitrogen − 50% oxygen × 1 h) in a double-blind, cross-over design with nitrous oxide and placebo administered 1 week apart. Patients were required to have failed at least two adequate trials of antidepressant treatments in the current episode of MDD while having a baseline depression score on the HDRS above 18. The trial excluded patients with BP, psychosis, panic disorder, obsessive–compulsive disorder, personality disorders, and active substance use disorder within the past 12 months except for nicotine. Also excluded were individuals with acute medical illnesses, acute suicidal behavior and prior treatment with ketamine or ongoing ECT. Patients were required to remain on their routine antidepressant treatments for 4 weeks prior to and during the trial.

Patients enrolled in this study averaged 48 years of age, were 60% women, and had experienced a mean lifetime history of 19 years of MDD and eight documented antidepressant failures; four had failed ECT and three had failed VNS; thus, the patients studied with nitrous oxide mirrored the patients in the TRMD Clinic and involved individuals with a high degree of treatment refractoriness (25). Interestingly, 10 patients had a history of migraine headaches and 4 had a history of corrected hypo­thyroidism. Significant improvement in depression ratings in this highly treatment-resistant cohort were observed at 24 h after nitrous oxide administration with a median reduction of 5.5 points on HDRS (95% CI −2.5 to −8.5 points), the primary endpoint for the study. Four patients were rated as showing a response to treatment (50% decrease in HDRS) and one of these individuals showed remission of MDD symptoms (HDRS < 7) compared to one patient with response to placebo and no placebo-treated patients with remission. MDD symptoms that were particularly sensitive to nitrous oxide included depressed mood, suicidal ideation, guilt, and psychological symptoms of anxiety. A significant subset of those patients experiencing an antidepressant response to nitrous inhalation maintained their response at 1 week. This latter feature confounded the cross-over study design because benefits were still observed at the time of the subsequent treatment. Nitrous oxide was well tolerated and side effects were short-lived following termination of nitrous administration. Side-effects included nausea and vomiting, headache and increased anxiety/panic, and necessitated shortening the duration of nitrous administration in 5 of the 20 patients (vs. none of the placebo administrations).

## Nitrous Oxide and NMDARs

How does nitrous oxide produce its effects? Studies in the late 1990s provided the first evidence that nitrous oxide is a non-competitive NMDAR antagonist. In these studies, nitrous blocked NMDAR-activated currents in cultured hippocampal neurons with an approximate half-maximal effective concentration (EC50) of 30–40% (95). Even at 80%, however, nitrous was only a partial inhibitor of NMDAR responses producing less than 70% block. Unlike ketamine, nitrous oxides effects were weakly voltage-dependent and did not display the kinetic effects on NMDAR currents expected of an open channel blocker, including having no effect on the decay of NMDAR-mediated synaptic currents (10). Nitrous oxide had minimal effects on GABA-A receptors, with very weak (~20%) potentiation observed at 80% nitrous. Weak effects were also observed on AMPA/kainate type glutamate receptors (non-NMDARs) with about 30% block of kainate currents (mediated mainly by AMPARs in this preparation) at 80% nitrous. Effects on AMPAR currents were not voltage dependent. Nitrous had small inhibitory effects on synaptic currents mediated by AMPARs but did not alter paired-pulse plasticity of these responses. This latter effect is important because it indicates that nitrous oxide is unlikely to have large presynaptic effects on glutamate transmission. Consistent with this, nitrous oxide failed to inhibit high voltage activated (HVA) calcium currents; HVA currents provide the calcium signal that triggers synaptic glutamate release.

The effects of nitrous oxide as an NMDAR antagonist in hippocampal neurons were consistent with histological changes observed in the cortex of rodents *in vivo*. Prior studies showed that NMDAR antagonists, including ketamine, produce vacuoles in neurons in the posterior cingulate cortex following systemic administration (37). Nitrous oxide mimicked these effects (96). Similarly, NMDAR antagonists block glutamate-mediated excitotoxicity and nitrous oxide mimicked these effects as well. Thus, physiological and histological studies indicate that nitrous oxide has NMDAR antagonistic, neuroprotective, and neurotoxic actions.

## Nitrous Oxide and Brain Circuits

To date, few studies have examined how nitrous oxide alters neuronal network activity. In the CA1 region of rat hippocampal slices, acute nitrous perfusion resulted in disinhibition of population spike firing without significant effects on AMPAR-mediated synaptic potentials (97). The disinhibition was occluded by picrotoxin, a GABA-A receptor antagonist, suggesting that nitrous oxide altered local synaptic inhibition in the CA1 region. In these studies, the lack of effect of nitrous oxide on excitatory synaptic potentials in the CA1 Schaffer collateral pathways suggests that its actions may differ from ketamine (72). However, the nitrous oxide studies were done in hippocampal slices from juvenile rats and ketamine’s ability to enhance CA1 synaptic responses appears to be age-dependent and most prominent in slices from adult rodents (98). Nonetheless, the disinhibitory effects on CA1 function observed in young rodents is consistent with apparent changes in population spike firing observed with ketamine at the same age (78) and studies of ketamine in cortex (68).

There is limited information about the effects of nitrous oxide on human brain networks. Studies using quantitative electroencephalographic (EEG) recordings indicate that nitrous oxide dampens functional connectivity in superficial parietal networks with more widespread changes in frontal activity (99). Other work has found that nitrous oxide (at 20–40%) acutely decreases frontal slow wave (delta) activity, but this is followed by enhanced theta activity after drug washout (100). These EEG effects of nitrous differ from other general anesthetics. Studies examining the effects of nitrous on connectivity of brain networks underlying depression are lacking.

Few studies have directly linked nitrous oxide’s effects on NMDARs to specific behaviors. In the roundworm, *Caenorhabditis elegans*, Nagele and colleagues (101) found that nitrous oxide alters motor reversal behavior. These effects of nitrous were not observed in animals lacking functional NMDARs, but were present in worms with altered expression of non-NMDARs. Furthermore, nitrous oxide inhibited motor reversal behavior upon re-expression of NMDARs in the NMDAR mutant worms. At 70%, nitrous oxide had no effect on chemotaxis, feeding, defecation or rate of locomotion. Some evidence in mice with targeted deletion of the GluN1 NMDAR subunit indicates that NMDARs are involved in the sedating effects of nitrous oxide (102), although this finding has not been replicated in other studies and secondary effects on monoaminergic activity may be important for immobility in GluN1 knockout animals (103). Other work suggests that discriminative stimulus effects of nitrous oxide at least partially involve NMDARs (104).

## Effects of Nitrous Oxide on Voltage-Activated Calcium Channels

The analgesic effects of nitrous oxide have prompted studies examining whether the drug alters ion channels or signaling systems involved in pain processing other than NMDARs. Todorovic and colleagues (105) found that nitrous oxide is a weak inhibitor of low voltage activated (LVA, T-type) calcium channels, producing about 30% depression of LVA currents at 80% nitrous in rat sensory neurons. Interestingly, the ability of nitrous to block LVA currents is selective for the Ca_v_3.2 LVA subtype that is expressed in sensory neurons, with no effect on Ca_v_3.1. Other studies using site directed mutagenesis have found that effects on Ca_v_3.2 likely involve metal catalyzed oxidation (MCO) at histidine 191 (H191) (106, 107). MCO is a form of the redox modulation that is known to alter the function of multiple ion channels including LVA channels (108) and NMDARs (109). Consistent with this, Orestes et al. (106) showed that nitrous oxide generates reactive oxygen species (ROS) in the presence of iron, and metal chelators (that scavenge iron) and catalase (a ROS scavenger) attenuate effects of nitrous on LVA channels. Furthermore, mutant mice with targeted deletion of Ca_v_3.2 show diminished analgesia in response to nitrous oxide, indicating that this channel is relevant to peripheral analgesic effects of nitrous oxide *in vivo*. Similarly, EUK-134, an agent that mimics superoxide dismutase and catalase, also dampens nitrous oxide-induced analgesia (106). Nitrous oxide does not inhibit high voltage activated (HVA) calcium currents in sensory or hippocampal neurons (10, 105).

## Other Potential Mechanisms of Nitrous Oxide

**Figure 4:**
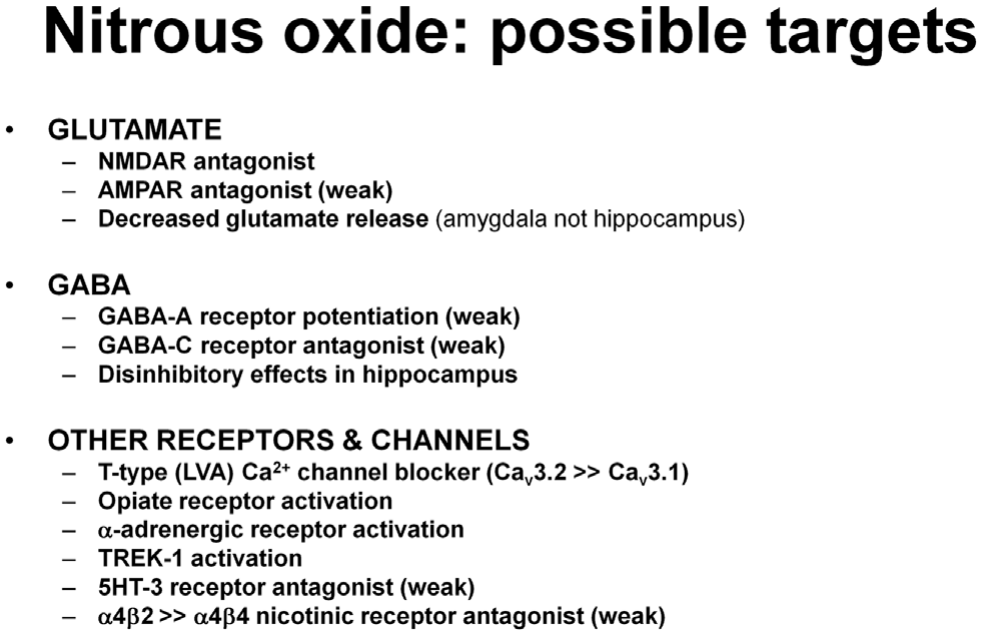
**The list includes various effects of nitrous oxide that could contribute to antidepressant and other CNS effects**. Supporting studies are described in the text.

Nitrous oxide has other effects that could contribute to its clinical and behavioral actions, particularly analgesia (Figure 4). These include the ability to activate the two pore-domain potassium channel, TREK-1 (110), as well as to weakly block GABA-C receptors and serotonin-type 3 receptors (111). Nitrous is also a partial inhibitor of certain nicotinic acetylcholine receptors, producing 40% inhibition of α4β2 nicotinic receptors but only 7% inhibition of α4β4 receptors (111). Other evidence indicates that nitrous oxide has opioid-like effects that could contribute to analgesia and perhaps to psychotropic effects (112–115). Nitrous oxide also has effects on brainstem adrenergic neurons and activation of α-adrenoceptors in brainstem and spinal cord contribute to antinociceptive effects in rats (116, 117).

It is difficult to compare the potency of nitrous oxide for effects on various targets across studies because complete concentration-response data are not available for many effects. Nonetheless, nitrous oxide blocks NMDARs by 50% at a concentration of 30%, making this one of its most potent effects (96). Effects on opiate receptors are also potent with 30% nitrous having analgesic actions equivalent to 10–15 mg morphine (118). Actions at T-channels (30% block at 80% nitrous) (105) and TREK-1 (30% activation at 80% nitrous) (110) are weaker.

In addition to analgesia and anesthesia, nitrous oxide is used clinically for anxiolytic effects. Consistent with this, acute nitrous oxide shows anxiolytic properties in the elevated plus maze (119), light-dark exploration (120), social interaction (121), and condition burying tests (122) that are used as animal models of anxiety. These effects have been observed during 15–30 min exposures to nitrous oxide at concentrations of 25–75%. How nitrous oxide produces these effects is not certain, although some evidence suggests a role for serotonin and GABA-A/benzodiazepine receptors (123) and the release of the endogenous messenger, nitric oxide (NO) (118, 124). Cyclic GMP, a messenger activated by NO, also appears to participate in the anti-anxiety effects of nitrous oxide in rodents (125). NO has presynaptic effects on glutamate release, and nitrous oxide dampens NMDAR-mediated synaptic responses in the amygdala by both postsynaptic and presynaptic actions (126). Zarate and Machado-Vieira (127) postulated that the ability of nitrous oxide to generate NO may be important in its antidepressant actions. Consistent with this, some synaptic and memory impairing effects of ketamine involve NO synthesis (78, 128).

## Ongoing Issues and Future Considerations

Studies examining the acute antidepressant effects of ketamine and other NMDAR antagonists are a growth area in psychiatry, providing inroads into what may be truly novel targets and treatments for MDD and TRMD (8, 88). The ability of these agents to rapidly dampen suicidal ideation leads to the intriguing possibility of administering ketamine-like drugs in emergency and inpatient settings while other antidepressant strategies are initiated. Despite encouraging results there are major challenges with the use of ketamine, including acute psychotomimetic and cognitive-impairing actions, along with abuse potential. It is also important to remain cognizant of the challenges that have plagued attempts to develop NMDAR antagonists as treatments for neurodegenerative conditions, including neurotoxic effects (37).

The recent pilot findings with nitrous oxide for TRMD are intriguing and indicate that mechanisms other than channel block for inhibiting NMDAR function may be effective for treating patients with severe and refractory mood disorders. Like ketamine, nitrous oxide appears to have rapid beneficial effects on suicidal ideation. Importantly, while nitrous oxide, like ketamine has abuse potential, it does not appear to share ketamine’s psychotomimetic or cognitive side effects, making it an attractive alternative for therapeutic development. Nitrous oxide is known, however, to impair methionine synthesis via inactivating effects on vitamin B12 (cobalamin), particularly following repeated or prolonged administrations (94). In the pilot antidepressant trial, a single 1 h administration of nitrous oxide did not alter vitamin B12 activity (11).

As is true of any pilot study, there are now many more questions than answers including questions that are relevant for ketamine (129). The pilot nitrous oxide study used a single dose and a single 1-h administration. The dose (50% nitrous oxide) was based on use in anesthesiology and dentistry, while the duration (1 h) was selected to mimic ketamine infusions for TRMD. Despite initial positive results, it is unclear whether either of these parameters is optimal or whether lower doses and altered durations of exposure would maintain or improve benefits while diminishing acute side effects and enhancing patient tolerability during nitrous inhalation (100). Similar to ketamine, it is unclear how long the benefits of nitrous oxide last and how to sustain the benefits. The pilot study was conducted in highly refractory TRMD subjects; whether nitrous oxide will have a different impact in less refractory patients requires further study. Similarly, we have no information about the response of MDD in the context of other psychiatric disorders including BP, anxiety disorders, personality disorders and substance use disorders, or whether nitrous oxide will be effective in acute inpatient settings. While nitrous oxide is relatively easy to use in outpatient settings such as dental offices, monitoring cardiovascular and respiratory parameters as well as recovery from sedation will be important for use in psychiatry. Potential neurotoxic effects of nitrous oxide (and ketamine) also must be considered with high doses and more prolonged administration (96).

Another major issue in studies of rapid antidepressants concerns how best to measure changes in depression. Current instruments (e.g., HDRS, MADRS, and BDI) are limited and query symptoms that are of longer duration and less amenable to acute change (e.g., sleep disturbances, appetite, and weight changes). Thus, the validity of these instruments when dealing with rapid changes requires further evaluation. Efforts using other instruments, perhaps including visual analog scales and/or ecological momentary assessments may be more appropriate going forward.

Similar to other drugs, including ketamine, nitrous oxide is not specific for NMDARs and alters the function of a number of other ion channels and proteins involved in neural signaling. Nonetheless, effects on NMDARs are among the most potent effects of this agent, making it likely that NMDAR inhibition contributes, at least in part, to clinical effects, just as it likely does for ketamine. Although the NMDAR effects of ketamine may be modulated by regional or temporal differences in agonist presentation, depolarization state of cells, and by presence of local endogenous allosteric regulators (63, 69), the effects of nitrous oxide do not involve ion channel block, particularly the longer-lived trapping block associated with ketamine-type agents (10). Thus, effects of nitrous oxide may be less prone to environmental conditions. Also, the acute effects of nitrous oxide are faster to reverse once administration is stopped.

Whether nitrous oxide’s effects on NMDARs involve redox modulation as they do at LVA (T-type) calcium channels (106) remains to be determined. If this is the case, it opens up the possibility of developing other agents that target this mechanism, although specificity of effects could be a major problem. Unlike ketamine, however, nitrous oxide is not metabolized endogenously to other moieties that are bioactive (67, 130, 131), and both ketamine and its metabolites affect targets other than NMDARs that contribute to sedation and CNS effects (132). Furthermore, in the United States ketamine is sold as a mixture of S- and R-enantiomers; the S-enantiomer (which is what is used in Europe) is more potent but the R-enantiomer has biological activity (133). Thus, despite enthusiasm over the early work on ketamine and pilot results from nitrous oxide, it is premature to conclude that effects on NMDARs are the sole mechanism for antidepressant actions (129).

The clinical trial with nitrous oxide was based on the evolving literature on ketamine. There are other NMDAR antagonists used clinically that could also be potentially developed for therapeutic purposes in psychiatry. Examples include the gaseous anesthetic, xenon (111, 134), the common intoxicant, ethanol (alcohol) (135), and the cough suppressant, dextromethorphan. Whether any of these agents would have antidepressant efficacy, particularly following acute systemic administration, is uncertain. Based on the lack of apparent antidepressant efficacy of memantine (91), however, it seems unlikely that all NMDAR antagonists will be useful as antidepressants. Nonetheless, understanding why memantine or these other agents do or do not have significant antidepressant effects may help clarify key mechanisms underlying psychotropic actions. In fact, preclinical studies with memantine have already pointed to differences in the ability of this agent to modulate spontaneous synaptic activity and to enhance synaptic efficacy in the rodent hippocampus relative to ketamine (93). Differences in pharmacokinetics and route of administration may also be key variables. Other considerations, include the fact that effective doses of ketamine and nitrous oxide are subanesthetic (65), indicating that at the effective doses, these agents only partially inhibit NMDAR currents, resulting in a significant population of NMDARs that remain unblocked. Studies in rodents indicate that anesthetic doses of ketamine that block a higher percentage of NMDARS also block both antidepressant actions in rodents and synaptic effects in neocortex (71). In hippocampus, complete block of NMDARs during ketamine administration (or high concentrations of ketamine alone) prevents synaptic and metaplastic effects of the drug in the hippocampus (78, 136). Whether similar effects occur with nitrous oxide and other NMDAR antagonists is unknown.

## Summary

Evolving work on NMDAR antagonists supports their importance as novel targets for therapeutic development in neuropsychiatry. Ongoing studies of ketamine and early results with nitrous oxide are encouraging. While neither of these agents is a selective NMDAR antagonist, it is clear that NMDAR inhibition plays a role in their behavioral effects. Thus, nitrous oxide is another chapter in the NMDAR depression story and has the potential to open new avenues for rapid therapeutic effects against severe and refractory forms of MDD.

## Author Contributions

All authors participated in the concept and writing of this manuscript. All authors approved the final version of the manuscript.

## Conflict of Interest Statement

Charles F. Zorumski is a member of the Scientific Advisory Board of Sage Therapeutics. Peter Nagele has a patent pending on the use of nitrous oxide for treatment of depression. There are no other competing financial interests. Sage Therapeutics did not fund this research and was not involved in the conduct of this research.

## Abbreviations

AMPA, α-amino-3-hydroxy-5-methyl-4-isoxazolepropionic acid; BDI, Beck depression inventory; BDNF, brain-derived neurotrophic factor; BP, bipolar disorder; CNS, central nervous system; CRPS, chronic regional pain syndrome; CBT, cognitive-behavioral therapy; DSM-V, Diagnostic and Statistical Manual of Mental Disorders-Fifth Edition; EMA, ecological momentary assessments; ECT, electroconvulsive therapy; EEG, electroencephalogram; fMRI, functional magnetic resonance imaging; GABA, γ-aminobutyric acid; EC50, half-maximal effective concentration; HDRS, Hamilton Depression Rating Scale; HVA, high voltage-activated calcium currents; IPT, interpersonal therapy; LTD, long-term depression; LTP, long-term potentiation; LVA, low voltage activated calcium currents; MADRS, Montgomery–Asberg Depression Rating Scale; MAOIs, monoamine oxidase inhibitors; MCO, metal catalyzed oxidation; MDD, major depressive disorder; mTOR, mammalian target of rapamycin; NO, nitric oxide; NMDARs, *N*-methyl-d-aspartate receptors; PCP, phencyclidine; PTSD, post-traumatic stress disorder; ROS, reactive oxygen species; rTMS, repetitive transcranial magnetic stimulation; SSRIs, selective serotonin reuptake inhibitors; SNRIs, serotonin and norepinephrine uptake inhibitors; TCAs, tricyclic antidepressants; TRMD, treatment-resistant major depression; VAS, visual analog scales; VNS, vagus nerve stimulation.
